# Clinical insight among persons with schizophrenia spectrum disorders treated with amisulpride, aripiprazole or olanzapine: a semi-randomised trial

**DOI:** 10.1186/s12888-023-04981-9

**Published:** 2023-06-29

**Authors:** L.A Stabell, E. Johnsen, R. A Kroken, E.M. Løberg, A. Blindheim, I. Joa, S.K. Reitan, M. Rettenbacher, P. Munk-Jørgensen, R. Gjestad

**Affiliations:** 1 Division of Psychiatry and NORMENT Centre of Excellence, Haukeland University Hospital, Bergen, Norway; 2 Department of Clinical Medicine, University of Bergen, Bergen, Norway; 3 Faculty of Psychology, Department of Clinical Psychology, University of Bergen, Bergen, Norway; 4 Department of Mental Health, St. Olav University Hospital, Trondheim, Norway; 5 Department of Mental Health, Faculty of Medicine and Health Sciences, Norwegian University of Science and Technology, Trondheim, Norway; 6 Department of Psychiatry, Psychotherapy and Psychosomatics, Medical University of Innsbruck, Innrain 52, Innsbruck, Austria; 7 Network for Clinical Research in psychosis, TIPS, Stavanger University Hospital, Stavanger, Norway; 8 Faculty of Health, Network for Medical Sciences, University of Stavanger, Stavanger, Norway; 9 Department of Psychiatry, University of Southern Denmark, Odense, Denmark; 10 Centre for Research and Education in Forensic psychiatry, Haukeland University Hospital, Bergen, Norway; 11 Research Department, Sandviken sykehus, Haukeland University Hospital, P. Box 1400, Bergen, 5021 Norway

**Keywords:** Antipsychotics, Antipsychotic-naïve, Insight, Effectiveness, Longitudinal

## Abstract

**Background:**

Antipsychotic treatment may improve clinical insight. However, previous studies have reported inconclusive findings on whether antipsychotics improve insight over and above the reduction in symptoms of psychosis. These studies assessed homogeneous samples in terms of stage of illness. Randomised studies investigating a mixed population of first- and multiepisode schizophrenia spectrum disorders might clarify this disagreement.

**Methods:**

Our data were derived from a pragmatic, rater-blinded, semi-randomised trial that compared the effectiveness of amisulpride, aripiprazole and olanzapine. A sample of 144 patients with first- or multiepisode schizophrenia spectrum disorders underwent eight assessments during a 1-year follow-up. Clinical insight was assessed by item General 12 from the Positive and Negative Syndrome Scale (PANSS). We analysed latent growth curve models to test if the medications had a direct effect on insight that was over and above the reduction in total psychosis symptoms. Furthermore, we investigated whether there were differences between the study drugs in terms of insight.

**Results:**

Based on allocation analysis, all three drugs were associated with a reduction in total psychosis symptoms in the initial phase (weeks 0–6). Amisulpride and olanzapine were associated with improved insight over and above what was related to the reduction in total psychosis symptoms in the long-term phase (weeks 6–52). However, these differential effects were lost when only including the participants that chose the first drug in the randomisation sequence. We found no differential effect on insight among those who were antipsychotic-naïve and those who were previously medicated with antipsychotics.

**Conclusions:**

Our results suggest that antipsychotic treatment improves insight, but whether the effect on insight surpasses the effect of reduced total psychosis symptoms is more uncertain.

**Trial registration:**

ClinicalTrials.gov Identifier: NCT01446328, 05.10.2011.

## Introduction

Clinical insight, which is defined as being aware of having a mental disorder and having the ability to recognise symptoms of the disorder and the need for treatment [1], is impaired in up to 80% of patients with schizophrenia [2]. It has been documented in both first- and multiepisode schizophrenia [3, 4], as well as in active and more stable phases of the disorder [5, 6]. Impaired clinical insight has small to modest correlations with other symptoms of psychosis and depression [1, 7] and neurocognitive dysfunction [3, 4, 8]. It has been suggested that changes in insight may be neglected as an effect of treatment due to improvement in other symptoms [5]. Moreover, persistent impairment in insight has also been reported [6, 9]. Impaired clinical insight is associated with poorer treatment adherence [10], which makes it a major obstacle for improvement [11, 12] with secondary consequences, such as relapse and readmission to the hospital [13] and even increased mortality [14, 15]. Thus, testing whether antipsychotic medication has a direct effect on insight and whether there are differential effects among antipsychotic medications is important to guide clinical practice.

Antipsychotic drug therapy is a cornerstone in the treatment of schizophrenia spectrum disorders [16]. However, variance in efficacy among antipsychotics has been demonstrated [17], and it is less common that studies report the effects of antipsychotics on single symptoms [16, 18]. Some studies have examined the specific effect of antipsychotic medication on insight [19–23], and the relation between improved insight and overall symptom reduction has been demonstrated [19, 20, 24]. Mattila and colleagues used databases from 14 randomised, placebo-controlled trials to investigate the efficacy of antipsychotics on insight [24]. They detected no differential effects among the investigated drugs after 6 weeks of usage, but antipsychotic treatment led to a small improvement in insight through improvement in other psychosis symptoms. There are two large studies investigating different antipsychotics and how they influence clinical insight. The European First-Episode Schizophrenia Trial (EUFEST) [5] showed that antipsychotics improve insight over and above improvements in other symptoms and differential effects among the investigated antipsychotics. The Clinical Antipsychotic Trials of Intervention Effectiveness (CATIE) [25] reported an inverse association between improvement of insight and illness severity. Both studies investigated homogeneous samples: first-episode and chronic-phase schizophrenia. This makes inference of the findings to the general population with schizophrenia spectrum disorders challenging, and the findings may not be valid in more heterogeneous samples representative of real-life clinical practice.

The stage of illness may influence the level of insight [26]. One study found first-episode psychosis patients to have more severe deficits in insight at admission but significantly better insight than multiepisode patients at discharge [27]. Additionally, a study with a combined sample of first- and multiepisode patients found improvements in insight to be more enduring among first-episode psychosis patients, whereas multiepisode patients had improved insight during inpatient treatment but returned to baseline levels after discharge [26]. However, few studies have investigated the effect of medication on insight over time in samples consisting of patients with both first- and multiepisode schizophrenia. As the stage of illness may also influence the effect of antipsychotics [15, 24], more longitudinal randomised studies representative of a clinically relevant mixed population are needed to establish stronger evidence of the relationship between insight, symptom reduction and the use of antipsychotic medication.

The present study reports results from the BeSt InTro trial, a pragmatic, semi-randomised, rater-blinded, head-to-head comparison between three first-line atypical antipsychotics: amisulpride, aripiprazole and olanzapine [28]. These antipsychotics were chosen due to their differing receptor affinities and the fact that they had not been previously compared head-to-head. Although the idea of pragmatic trials was introduced more than five decades ago [29], the BeSt InTro trial reignited the debate on pragmatic antipsychotic trials after being published in Lancet Psychiatry [30–32]. A recent review argues that after a drug is approved for the market, pragmatic trials add value, as they reflect real-life clinical decision-making [33].

The aim of this study was to examine the longitudinal effectiveness of amisulpride, aripiprazole and olanzapine on insight and whether these possible effects were over and above the effect of an overall reduction in psychosis symptoms. Based on the EUFEST study, we hypothesised that there will be differences among the three study drugs regarding their impact on insight. In addition, some will have an added effect beyond a change in psychosis symptoms. Furthermore, we anticipated that antipsychotic-naïve patients would experience a stronger effect on insight independent of the type of antipsychotic that was used.

## Material & methods

### Design

This is a study on a predefined secondary outcome from the Bergen-Stavanger-Innsbruck-Trondheim (BeSt InTro) Study, a multicentre, longitudinal, pragmatic, semi-randomised trial_1_. More elaborate details on the method can be found elsewhere [28]. The study was prospective with comprehensive assessment of symptoms, neurocognitive function and blood tests at baseline and weeks 1, 3, 6, 12, 26, 39 and 52. The BeSt InTro trial was scored as pragmatic on seven of the nine items of the PRECIS-2 tool (31).

### Sample

Four study sites, all located in major cities in Norway and Austria, recruited the participants. Inclusion took place from 20 October 2011 to 30 December 2016. Data collection was completed on 21 December 2017. Inclusion criteria were as follows: at least 18 years of age; ability to cooperate with oral treatment with antipsychotic medication; International Statistical Classification of Diseases and Related Health Problems (ICD-10) (WHO, 1994) diagnosis within the F20-F29 chapter; and a score of four or more on at least one of the following items of the Positive and Negative Syndrome Scale [34]: P1 delusions, P3 hallucinations, P5 grandiosity, P6 suspiciousness/persecution or G9 unusual thought content, indicating an acute phase of psychosis. Exclusion criteria were as follows: pregnant and lactating women, inability to understand the native language, organic psychosis, hypersensitivity to the active substances in the study drugs, or somatic contraindications.

### Procedure

Participants were randomised to a sequence of the three antipsychotic drugs. If the patient did not accept the first drug in the sequence due to having severe side effects or unsatisfactory results from its use prior to enrolment, the next antipsychotic was offered. The reason for rejecting the first drug in the sequence was noted. As this procedure is more flexible than traditional randomisation, the study is characterised as semi-randomised [28]. The study assessors were blinded to the randomisation, but it was open to the patient, the treating physician and the rest of the clinical staff. The treating physician was in charge of determining the dose and duration of treatment; therefore, it was not considered a protocol violation if the participant’s study drug was changed or terminated during the study period. Change from oral formulation to long-lasting injections of the study drugs, or use of doses outside the ranges defined by the Summaries of Product Characteristics classified as protocol violations. We measured the serum concentration of the chosen study drug at each assessment point, and the mean levels were generally within the therapeutic range for all three drugs throughout the study. Additional use of other antipsychotics was not allowed according to the protocol, except for overlap during antipsychotic drug switches.

### Measurements

Participants were considered antipsychotic-naïve if they stated they had never used antipsychotics before. This was verified by checking their medical records. The Positive and Negative Syndrome Scale (PANSS) [34] assessed psychopathology, including insight. The PANSS has good reliability and validity [35]. All assessors were trained and certified by the PANSS Institute in New York. The item G12 in the PANSS assesses the individual’s ability to recognise symptoms and the need for treatment, as well as the capacity to make judgements, hereafter referred to as insight. This item correlates highly with other more elaborate measures of insight in schizophrenia [36, 37]. Higher scores represent more impaired clinical insight. When reporting on total psychosis symptoms, we used the PANSS total minus item G12. This is referred to as the modified PANSS (mPANSS). Higher scores represent more symptoms.

### Statistics

Descriptive analyses and t tests were run in the software package SPSS statistics, version 24 [38]. For some PANSS assessments, information in eight items was missing. To compute sum scores, we used SPSS Expectation Maximisation (EM) to impute values in these variables [39]. As is typical in such studies, drop-out and intermittent missing data were considerable. Of the 144 patients, 44 had responses on all measurement occasions. The data coverage over the eight occasions were (%): 100, 90, 84, 70, 60, 48, 44 and 44. We used full information maximum likelihood (FIML) to use all available data. This assumes missingness to be randomly distributed (MAR) and represents a better strategy than using listwise deletion assuming missing completely at random (MCAR) [40]. A consequence of the MAR assumption is the unnecessity of exploring the relationships between missingness and observed variables.

We analysed change per week in insight and the mPANSS in a latent growth curve model [41]. When inspecting and analysing the data, we found a nonlinear pattern of change at the mean level, with considerable individual variation. For both the mPANSS and insight, most change took place during the first 6 weeks of the study; thereafter, the change was much less pronounced. Therefore, we tested two-piece models and quadratic functions. The final model specified linear and quadratic slopes capturing linear and nonlinear change over time in the two intervals. The first (linear) slope represented the interval from baseline to week 6 and will be referred to as the initial phase. The second slope was the quadratic function in this initial phase. The third slope represented the change in the 6–52-week interval, referred to as the long-term phase. We used maximum likelihood robust (MLR) to handle minor nonnormality in the outcome measures [40]. The level of statistical significance was set at α = 0.05, two-tailed. Model fit was evaluated using the chi-square significance test (should be > 0.05), Comparative Fit Index (CFI > 0.95), Tucker-Lewis Index (TLI > 0.95) and Root Mean Square Error of Approximation (RMSEA, mediocre fit: <0.10; fair fit: <0.08; close fit: <0.05) with a standard confidence interval (CI) of 90% [40]. To predict the amount of change and to better balance the number of estimated parameters with the sample size, the quadratic function was removed in the prediction model [40] .

After establishing level and change models for mPANSS and insight, levels of and changes in these variables were related to the three medication groups. These were each represented by two dummy variables: aripiprazole (D1 = 1, D2 = 0), olanzapine (D1 = 0, D2 = 1) and amisulpride (D1 = 0, D2 = 0), with the latter used as the reference drug due to its clean receptor profile. The model specified the predictive relationships between levels of and changes in mPANSS as outcomes on medications and levels of and changes in insight as endogenous outcomes on medications together with levels of and changes in mPANSS. In this way, levels of insight predicted by levels of mPANSS, changes in the first interval of insight by changes in the same interval of mPANSS, and a similar prediction in the second interval. Thus, this model has both direct relationships between medication and insight and indirect relationships via levels of and changes in mPANSS. Even if this is technically a mediation model, we see this model as a set of regressions. Instead of correlating medication and levels of and changes in mPANSS, which would give an ordinary regression model at the structural level, we specified a predictive direction between medications and the mPANSS intercept and slope factors. This direction is the only way these relationships could go. Even though antipsychotic medication is causally related to reduction in psychosis symptoms, this model does not assume that all included variables are causally related, as this would require biological, cognitive, behavioural, and contextual mechanism variables to be included. Our model contains only direct and indirect predictions. Accordingly, we use the term “effect” in the meaning of statistically effects, and not causal effects.

We followed the protocol that was used in the primary trial [28]. Our primary analysis was based on the first study drug in the random sequence, hereby referred to as analysis #1a. This analysis included subjects as per allocation. Our secondary analysis was s based on the drug chosen by the patient and physician, hereby referred to as analysis #2. According to the protocol, participants were not excluded from the study due to change or termination of the study drug during follow-up, and consequently, both models in analysis #1a and analysis #2 investigated the same number of participants. Post hoc we ran a sensitivity analysis based on only the participants that chose the first drug from the randomisation sequence, hereby referred to as analysis # 1b. Due to randomisation, the standard strategy is to constrain potential differences between the study drugs to zero at baseline (intercept). Additionally, we added the variable antipsychotic-naïve as a predictor, as well as the interaction terms D1 * naïve and D2 * naïve. This would show potential differences in results for patients who had never received antipsychotics before and patients who were previously medicated. Furthermore, we investigated duration of use in interaction with each study drug. These models of change, predictive models and sensitivity analyses on missing mechanisms were analysed in Mplus version 8.2 [42].

## Results

### Descriptive

**Fig. 1:**
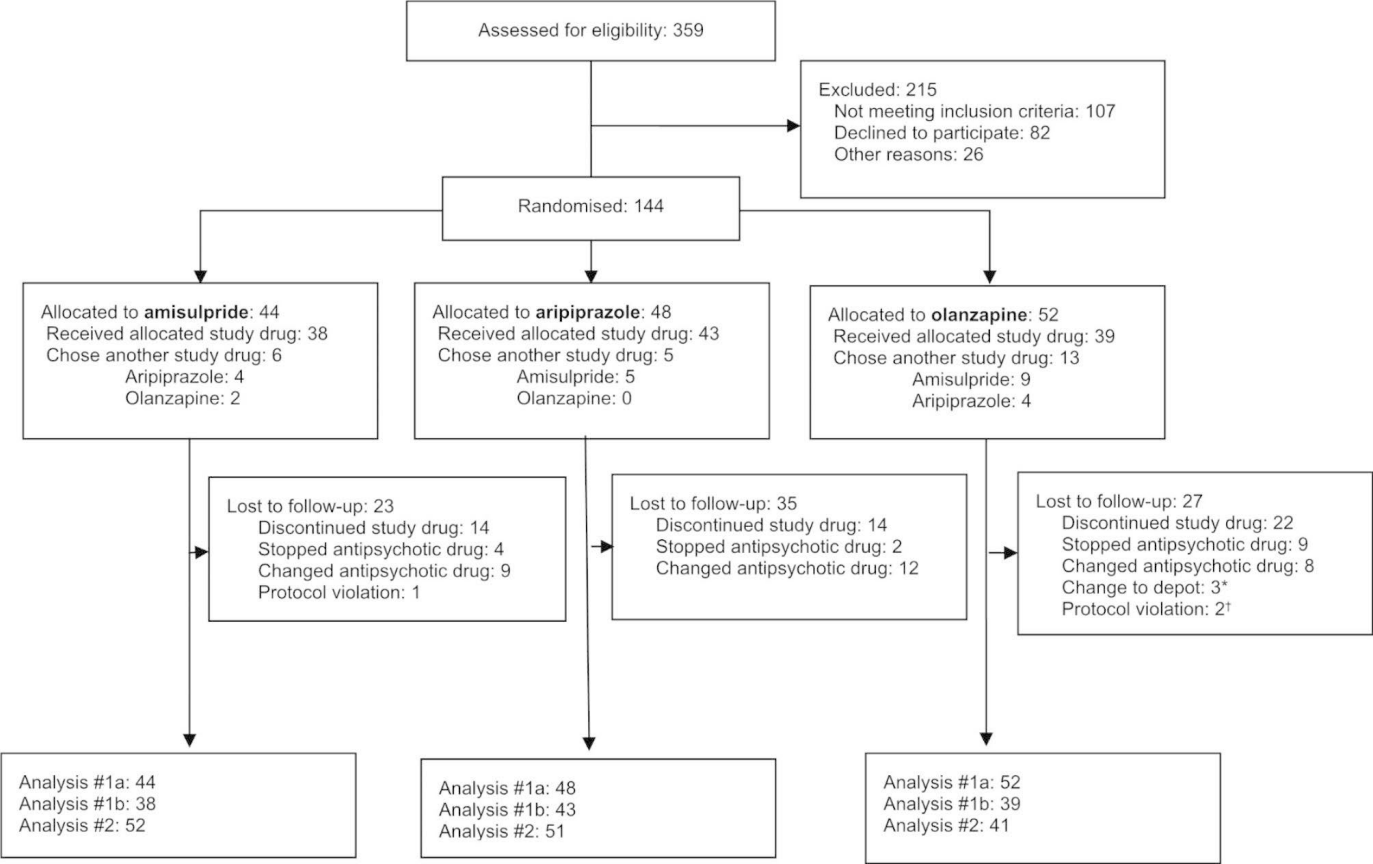
Flow of participants in the BeSt InTro study **Notes**: Lost to follow-up = explicit withdrawal from further participation in the study or not showing up at subsequent study visits; *Depot = long-acting formulation of study drug; †Protocol violation = use of dosage above upper limit according to the study protocol

Out of 359 eligible patients, 144 patients were included in the study. Reasons for exclusion were not meeting the inclusion criteria (n = 107), declined participation (n = 82) and being discharged before signing consent or being indecisive (n = 26). Figure 1 provides details regarding the flow of participants in the study. Because we are reporting on a secondary outcome of the BeSt InTro study, the flow diagram is identical to the previously published primary outcome [28]. Sample characteristics are shown in Table 1. We found no significant differences in sample characteristics at baseline between the three study drugs.

**Table 1:** Baseline characteristics of the sample in the BeSt InTro study

	All (N = 144)	Amisulpride (N = 44)	Aripiprazole (N = 48)	Olanzapine (N = 52)
Age	31.7 (12.7)	30.6 (11.7)	32.1 (13.1)	32.2 (13.3)
Men	93 (65%)	28 (64%)	32 (67%)	33 (63%)
White ethnicity	118 (82%)	39 (89%)	35 (73%)	44 (85%)
Years of education	12.3 (2.8)	12.7 (3)	11.9 (2.8)	12.2 (2.7)
Schizophrenia F20	84 (58%)	28 (64%)	27 (56%)	29 (56%)
Schizotypal F21	2 (1%)	1 (2%)	0 (0%)	1 (2%)
Delusional disorder F22	21 (15%)	4 (9%)	8 (17%)	9 (17%)
Acute and transient F23	18 (12%)	8 (18%)	3 (6%)	7 (13%)
Schizoaffective F25	10 (7%)	3 (7%)	5 (10%)	2 (4%)
Other nonorganic F28	1 (1%)	0 (0%)	1 (2%)	0 (0%)
Unspecified nonorganic F29	8 (6%)	0 (0%)	4 (8%)	4 (8%)
Antipsychotic-naïve	56 (39%)	16 (36%)	23 (48%)	17 (33%)
PANSS G12	3.4 (1.2)	3.4 (1.2)	3.7 (1.1)	3.2 (1.3)
mPANSS total	75.0 (15.5)	76.7 (18.4)	72.9 (13.1)	75.6 (15.0)
PANSS positive	21.2 (4.8)	21.4 (4.8)	21.3 (4.9)	21 (4.7)
PANSS negative	17.8 (6.1)	18.2 (7)	17.2 (5.6)	18.1 (5.8)
PANSS general - G12	36.0 (8.3)	37.0 (10.2)	34.4 (7.0)	36.5 (7.7)
CDSS	6.7 (5.1)	7.6 (5.7)	5.4 (4.5)	7.1 (5.1)
CGI	5.0 (0.8)	5.1 (0.9)	4.9 (0.7)	5.0 (0.8)
GAF	35.8 (9.3)	36 (9.6)	36 (9.6)	35.5 (8.8)

Abbreviations: N = number in the total sample; number in () is standard deviation, unless otherwise specified; PANSS: Positive and Negative Syndrome Scale; PANSS G12: General item 12 assessing insight; CDSS: Calgary Depression Scale for Schizophrenia; CGI: Clinical Global Impression Scale; GAF: Global Assessment of Functioning Scale-split version

### Levels of and changes in insight and the mPANSS

Table 2 shows the levels of and changes in insight and the mPANSS. At the mean level, both insight and total symptom burden improved most in the initial phase, and the improvement continued in the long-term phase. At baseline, the mean insight score was 3.36 (SD 0.92, range 1–6), and the mean mPANSS score was 74.54 (SD 14.28). The mean change in insight and the mPANSS was different over time, with a curvilinear pattern. In addition, there were individual differences among the participants around the mean changes over time. At the individual level, the baseline level of impaired insight did not influence how much insight improved in the initial phase (*r* = -0.29, p = 0.315). Linear change in the mPANSS in the initial phase was related to the baseline level (*r* = -0.44, p = 0.015), with a stronger reduction among those with higher mPANSS baseline levels. The evaluation of how well the statistical models fit the observed data indicated a good fit for the insight: χ^2^ = 35.20, df = 26, p = 0.107, CFI = 0.966, TLI = 0.963, RMSEA = 0.050, RMSEA_CI_ = 0.000-0.088, and RMSEA close fit = 0.47. The mPANSS model showed a satisfactory fit (χ^2^ = 44.74, df = 23, p = 0.004, CFI = 0.935, TLI = 0.921, RMSEA = 0.081, RMSEA_CI_ = 0.044–0.116, and RMSEA close fit = 0.76).

**Table 2:** Estimated level and change in insight and modified PANSS.

		Insight					mPANSS			
		*Mean*	*P* ^*a*^	*SD*	*P* ^*b*^		*Mean*	*P* ^*a*^	*SD*	*P* ^*b*^
Baseline		3.36		0.92	< 0.001		74.54		14.28	< 0.001
Change 0–6 w		-0.24	< 0.001	0.56	0.007		-7.28	< 0.001	7.06	0.002
Curvilinear 0–6 w		0.02	0.111	0.09	0.006		0.73	< 0.001	0.88	0.017
Change 6–52 w		-0.01	0.007	0.02	0.050		-0.16	< 0.001	0.25	0.050

PANSS: Positive and Negative Syndrome Scale

Insight: Item G12 from the PANSS

mPANSS: PANSS total score minus item G12

w: Weeks

SD: Standard Deviation

P ^a^: P value for means

P ^b^: P value for SD

### Analysis #1a

**Fig. 2:**
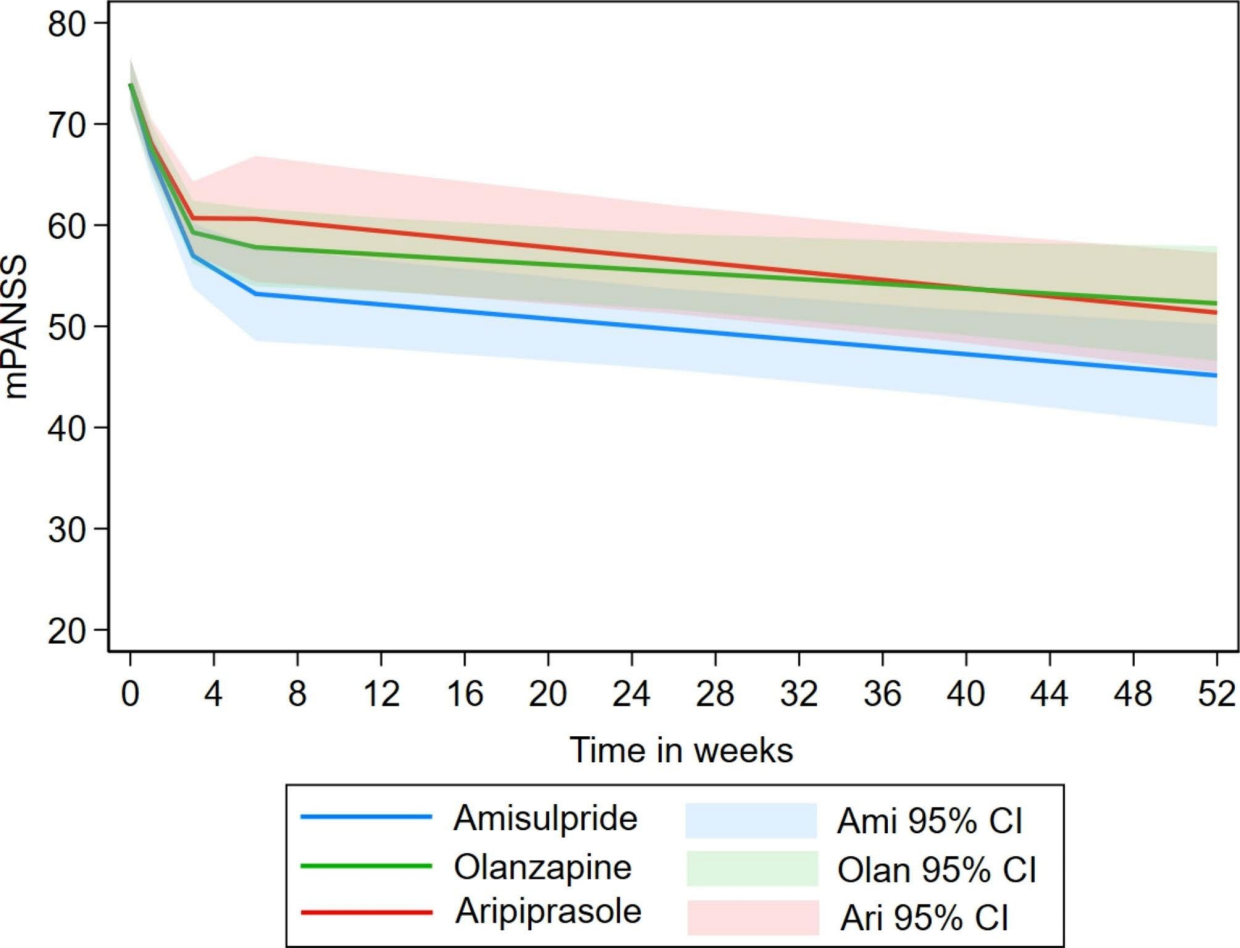
Pattern of estimated change in mPANSS among the three study drugs based on allocation in analysis #1a

This analysis was based on the first drug in the randomisation sequence, defining the following randomisation groups: amisulpride (n = 44), aripiprazole (n = 48) and olanzapine (n = 52). All study drugs showed relatively similar patterns of change in mPANSS in the initial phase and in the long-term phase (Fig. 2; Table 3), with reduction for the amisulpride group and similar reductions for the two other medication groups. The estimate for aripiprazole showed less reduction in the first phase compared to amisulpride as the referencegroup; however, the difference was observed ‘at trend level’ (p = 0.058).

**Fig. 3:**
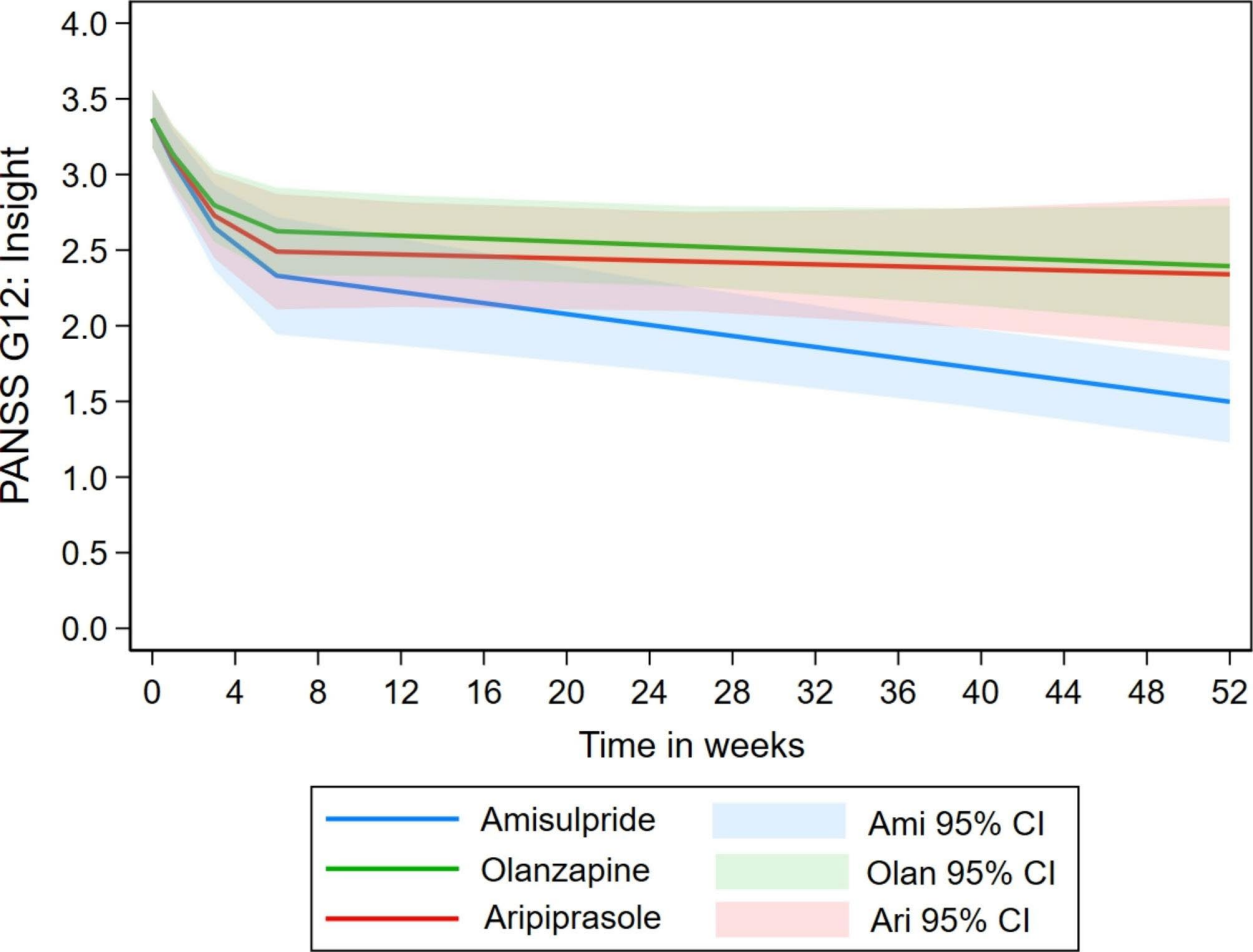
Pattern of estimated change in insight item G12 among the three study drugs based on allocation in analysis #1a

Regarding changes in insight, no medication differences were found in the initial phase. In the long-term phase, the amisulpride group showed a statistically significant reduction. Aripiprazole, on the other hand, showed statistically significant less reduction, showing that this group did not reduce their score (p = 0.366). Lastly, the olanzapine group did also not show reduction (p = 0.889), but this difference from the reference group was marginally beyond the 0.05 threshold (p = 0.075). These results are presented in Fig. 3. Although the figure visually indicates that amisulpride had a stronger reduction than olanzapine, the statistical differences between these two groups was not significant. The estimated mean change showed no changes for aripiprazole and olanzapine.

**Table 3:** Prediction of changes in mPANSS and Insight based on randomisation. Predictors are medications and changes in mPANSS. Baseline relations between mPANSS and Insight not given

		Changes in mPANSS						Changes in Insight				
		Initial phase			Long-term phase			Initial Phase			Long-term phase	
*Analysis #1a*		*b*	*p*		*b*	*p*		*b*	*P*		*b*	*p*
Amisulpride (*α*)		-7.88	< 0.001		-0.18	0.004		0.05	0.610		-0.01	0.033
Aripiprazole (*b1*)		1.24	0.058		-0.03	0.767		-0.03	0.472		0.02	0.030
- mean change		-6.64	< 0.001		-0.20	0.002		0.02	0.811		0.01	0.366
Olanzapine (*b2*)		0.77	0.144		0.06	0.545		0.01	0.710		0.01	0.075
- mean change		-7.11	< 0.001		-0.12	0.063		0.06	0.473		0.00	0.889
mPANSS change I								0.05	< 0.001			
mPANSS change L											0.05	0.001
Difference *b1-b2*		0.47	0.446		0.08	0.371		0.04	0.189		0.01	0.452
*Analysis #1b*												
Amisulpride (*α*)		-7.40	< 0.001		-0.19	0.006		0.04	0.714		-0.01	0.423
Aripiprazole (*b1*)		0.97	0.160		0.01	0.901		-0.02	0.641		0.01	0.195
- mean change		-6.43	< 0.001		-0.18	0.009		0.02	0.841		0.01	0.558
Olanzapine (*b2*)		0.80	0.183		0.03	0.772		-0.01	0.714		0.02	0.041
- mean change		-6.60	< 0.001		-0.17	0.004		0.03	0.765		0.01	0.206
mPANSS change I								0.05	< 0.001			
mPANSS change L											0.06	0.038
Difference *b1-b2*		0.17	0.790		0.02	0.872		0.01	0.768		0.01	0.610

Analysis #1a: Based on first drug in the randomisation sequence (n = 144)

Analysis #1b: Based on the participants that chose the first drug in the randomisation sequence (n = 120)

α: intercept level; *b1* and *b2* are regression estimates showing differences in changes from the reference group (amisulpride)

I: Initial phase

L: Long-term phase

Mean change: estimated absolute changes for the aripiprazole and olanzapine groups

The results also show decrease in mPANSS to be related to increase in insight in both intervals. The indirect relations between the medications and insight via mPANSS changes in the initial phase (b_indirect_ = -0.36, p < 0.001) and the long-term phase were both statistically significant (b_indirect_ = -0.01, p = 0.013). This means that increases in insight are associated with a reduction in mPANSS.

### Analysis *#1b*

This analysis is a restriction of analysis 1a, only including the participants that chose the first drug in the randomisation sequence: amisulpride (n = 38), aripiprazole (n = 43) and olanzapine (n = 39). The results from model 1b showed no similar improvements of insight in the long-term phase for the amisulpride as detected in analysis #1a. However, a worsening in insight was found for the olanzapine group. Other results were found equal to the ones found in model 1a.

### Analysis *#2*

These analyses were based on the study drug actually chosen by the patient and the physician, resulting in the following groups: amisulpride (n = 52), aripiprazole (n = 51) and olanzapine (n = 41). The only significant finding was an indirect association in the initial phase (b = -0.34, p < 0.001; long-term phase: b = -0.01, p = 0.093; relation between change in insight and change in mPANSS: b = -0.001, p < 0.001), meaning improved insight was associated with reduced total psychosis symptoms. In this model, we found no significant differences among the three study drugs on insight (initial phase: α_Ami_ = 0.05, p = 0.572; b_Ari_ = -0.00, p = 0.967; b_Olan_ = -0.00, p = 0.571 and long-term phase: α_Ami_ = -0.01, p = 0.087; b_Ari_ = 0.02, p = 0.054; b_Olan_ = 0.01, p = 0.096). In both predictive models, baseline insight was predicted by the baseline mPANSS (b = 0.05, p < 0.001), meaning that higher total psychosis symptoms were associated with higher levels of impaired insight.

### Interaction analysis

We included the duration of use of the study drug and being antipsychotic-naïve as predictors of both insight and the mPANSS. The only significant finding was analysis #2 displaying a worsening of psychosis symptoms for antipsychotic-naïve patients using aripiprazole in the long-term phase (b = 0.37, p = 0.045). However, no differential relationships were found regarding changes in insight.

## Discussion

This is the first study to investigate differential effects of antipsychotics on insight in a mixed sample of antipsychotic-naïve and previously medicated patients. All study drugs improved insight through the overall reduction in psychosis symptoms. For amisulpride and olanzapine, the improvements in insight exceeded this overall reduction in the analysis based on the first drug in the randomisation sequence. However, these differential effects were lost when only including the participants that chose the first drug in the randomisation sequence, and in the analysis based on the drugs that were chosen by the patient and their treating physician. We found no differences regarding effectiveness on insight among those being antipsychotic-naïve and those previously medicated with antipsychotics.

We found that the level of insight was associated with symptom severity at baseline. This is in accordance with findings reported by Gharabawi et al. [7]. Furthermore, all study drugs were related to improvement in insight through the overall reduction in psychosis symptoms. This association is supported by previous studies [24, 25], suggesting that antipsychotic treatment may be effective in improving insight regardless of the type of medication. Unfortunately, pharmacological studies were not included in a recent systematic review and meta-analysis on treatment effects on insight, mainly due to selected age criteria [42]. Poor adherence may reduce the effect of antipsychotics, and it is linked to impaired insight, both for first- and multiepisode schizophrenia [43, 44]. However, studies ensuring adherence through treatment with antipsychotic long-acting injections support the relationship between reduction in PANSS total scores and improved insight [7, 9, 45]. Nevertheless, other treatment factors in addition to pharmaceutical treatment may also improve insight [42], making it challenging to declare a causal relationship between the use of antipsychotics and improved insight.

Our findings are in line with the EUFEST study, as both studies detected an association between change in insight and decrease in other psychosis symptoms, as well as differential effects among antipsychotics [5]. The pattern of change is similar, as both studies report a two-phase process. However, the timeframes were different in the initial phases. The first phase was from baseline to 3 months in the EUFEST study, whereas the initial phase in the present study lasted 6 weeks from baseline. The EUFEST study found no significant improvement in insight beyond the initial 3 months. This differs from our findings of improvements in insight beyond improvements in other psychosis symptoms in the long-term phase for amisulpride and olanzapine. One reason the EUFEST study found differential effects in the initial phase may relate to an overlap of 6 weeks with our long-term phase. Our findings of antipsychotics having only an indirect relation to insight during the first 6 weeks of treatment are in line with findings from Mattila and colleagues [24]. The differential effects detected in our study were found in the long-term phase. One explanation of this delay may be that understanding and acknowledging one’s disease is a process that requires alteration of one’s beliefs over time [3]. In light of the “psychological denial model” [46], claiming poor insight is a defence mechanism to cope with the distress of the psychosis, it is plausible to assume it will take some time to accept the circumstances, yet alone admit them to a therapist. In addition, the antipsychotic effect occurred later on complex neurocognitive processes, such as problem solving and abstraction, than on more basic processes, such as reaction time and attention [47]. Furthermore, increased insight might be due to psychotherapy [48] and psychoeducation. A recent meta-analysis investigating treatment effects on insight in psychotic disorders reported a medium no significant effect size of psychoeducational interventions [42]. In addition, one randomised study reported that insight improved more in the group that received both medication and psychosocial interventions compared to those assigned to pharmacotherapy alone [49]. Unfortunately, we had no information on what type or frequency of psychotherapy and psychoeducation the patients received; therefore, we cannot dismiss the contribution of such treatment effects in our sample. However, one can speculate if the frequency of therapy may have influenced the chances of switching antipsychotic treatment due to a lack of response or severe side effects. This may have ensured a more stable medication usage and, in combination with other treatments, may have further improved insight.

Our study also included participants without impaired insight in the analysis, in line with the 14 studies included in the paper by Mattila [24]. In contrast, the EUFEST study included participants with a score of two or more on G12. This affected their baseline levels and increased the potential for improved insight. Consequently, the means at baseline were lower in our study compared to the EUFEST study: 3.36 and 4.03, respectively. However, at the endpoint of the initial phase of the EUFEST study, the score was closer to ours at 6 weeks (2.32 and 2.48, respectively). The inclusion of participants without impaired insight strengthens the representability of our findings.

The EUFEST study that investigated first-episode schizophrenia detected differential effects after 6 weeks [5]. Therefore, we expected those who were antipsychotic-naïve to have a stronger effect on insight compared to those previously medicated with antipsychotics. However, this finding was not replicated in our study. One explanation may be that the EUFEST study used stricter inclusion criteria of a G12 score of two or more. Furthermore, we classified participants as antipsychotic-naïve if they had never used antipsychotics previously. Based on these two differences, one can argue that the antipsychotic-naïve patients in our sample represent a different population of first-episode schizophrenia patients than the EUFEST study. Surprisingly, the only association of being antipsychotic-naïve in the present study was a worsening of psychosis symptoms in the long-term phase among those using aripiprazole. However, we detected differential effects on insight among the antipsychotics in the entire sample in the long-term phase. Consequently, one could speculate if repeated assessments over a longer time period were necessary to detect differences among antipsychotics on insight in multiepisode schizophrenia patients. The lack of statistical findings may also be a result of low statistical power.

The differential effects were only detectable in analysis #1a. A possible reason may be that 24 participants chose a drug other than the first study drug in the random sequence. Consequently, we ran a sensitivity analysis focusing on the participants that chose the first drug in the sequence. Losing the differential effects when reducing the sample from 144 to 120 in analysis #1b indicate that our sample is too small to detect stable effects on insight that surpasses the effect of reduced total psychosis symptoms. Analysis #2 reflects the drugs that were chosen by the participants and their treating physicians. As the randomisation is no longer valid, these analyses are more prone to selection bias. Taken together, these results of all analyses would have been more solid if they were similar. This discrepancy does therefore not support a direct link between specific medications and changes in insight.

We had considerable attrition throughout the study. This was not surprising, as dropout is a known challenge in antipsychotic intervention studies [50], and our rate was similar to that in other studies [51]. The maximum likelihood estimator handled the attrition in the analysis under the assumption that missingness was randomly distributed, which was supported by analyses of missingness [28]. Thus, the chance of attrition bias was reduced.

Among the design limitations, we note that we did not include a placebo control group or an unmedicated group. This ruled out the investigation and comparison of natural fluctuations in insight, other symptoms, and their interaction. However, antipsychotic drug treatment is evidence-based and highly recommended in treatment guidelines [50, 52–54]. Henceforth, such a drug-free control group would be at odds with the pragmatic design aiming to mimic usual practice and has been considered ethically problematic. Nevertheless, small RCTs with nonantipsychotic control groups have recently been conducted in Australia [55] and the UK [56, 57]. Furthermore, all patients were in the acute phase of psychosis when included in the study. Although we have reported that most of the effect on insight was related to symptom reduction, we did not analyse changes in PANSS scores according to the symptom remission criteria proposed by Andreasen et al. [58]. We can therefore not claim that they reached or obtained remission.

Insight was only measured by item G12 from the PANSS, which is limited to evaluation by a clinician on a seven-point scale. Other more elaborate assessments of insight may have detected more detailed differential effects among the drugs. However, G12 correlates highly with more amplified assessments [37]. Furthermore, other possible factors, such as social functioning, depression and cognition, could have affected the results. These factors were not included in our analyses due to model complexity and sample size. A larger sample would also have given us the opportunity to explore generalizability across diagnoses, age levels or sex, which may be needed to understand more of the complexity of insight.

## Conclusion

In summary, the use of antipsychotics improved insight through a reduction in total psychosis symptoms. Our results of differential relationships among the three antipsychotic drugs investigated were ambiguous. This discrepancy does therefore not support a direct link between specific medications and changes in insight in our mixed sample. Future research in larger mixed samples may be able to shear some light on this matter.

## Data Availability

The datasets generated during and/or analysed during the current study are not available. According to Norwegian law, data sharing requires approvals from the regional Committees for Medical and Health Research Ethics and from the Data Protection Officer at Haukeland University Hospital on the basis of specific research proposals. Requests can be addressed to Professor Erik Johnsen, erik.johnsen@helse-bergen.no.

## Abbreviations

ICH: International Council on Harmonisation of Technical Requirements for Registration of Pharmaceuticals for Human Use
PANSS: Positive and Negative Syndrome Scale PANSS
EUFEST: The European First-Episode Schizophrenia Trial
CATIE: Clinical Antipsychotic Trials of Intervention Effectiveness
BeSt InTro: Bergen-Stavanger-Innsbruck-Trondheim Study
mPANSS: Modified PANSS
EM: Expectation maximisation
FIML: Full information maximum likelihood
MAR: Missing at random
MCAR: Missing completely at random
MLR: Maximum likelihood robust
CFI: Comparative fit index
TLI: Tucker-Lewis index
RMSEA: Root mean square error of approximation
RMSEAci: Root mean square error of approximation confidence interval
SD: Standard deviation
